# Prevalence of Autism Spectrum Disorder Among Ethnic Minority Groups in the UK, Sweden and the Netherlands: A Systematic Review

**DOI:** 10.1192/bjo.2026.11120

**Published:** 2026-06-30

**Authors:** Rahul Roy, Cornelius Ani, Shereen Haffejee

**Affiliations:** Surrey and Borders Partnership NHS Foundation Trust, Leatherhead, United Kingdom

## Abstract

**Aims:**

The prevalence of Autism Spectrum Disorder (ASD) varies across populations. Research shows that ethnicity and migration status may influence when and how ASD is diagnosed. Understanding ethnic differences in the prevalence of ASD and any associated drivers is important to ensure fair access to assessment and support. This review explores how ethnicity and migration background may influence ASD identification by examining theprevalence and incidence of ASD among ethnic minority groups in the UK, Sweden and the Netherlands. These countries have sizeable immigrant and minority populations. The review also compares patterns of ASD classification and diagnostic subtypes across these countries.

**Methods:**

We conducted a systematic review of epidemiological studies that reported ASD prevalence or incidence by ethnicity, parental country of birth, or migration status in these countries. We searched MEDLINE, Embase, PsycINFO, CINAHL and PubMed, along with grey literature including Google Scholar. Reference lists of included studies were also screened for relevant studies.

Eight studies published between 1987 and 2025 met inclusion criteria. They drew on large population datasets such as education records, health and psychiatric registers, child health services and clinical service data. We summarised the findings with attention to study design, diagnostic criteria and methods used to identify ASD cases and patterns of ethnic differences.

**Results:**

Findings differed across countries and diagnostic subtypes. In the UK, most studies reported higher recorded ASD prevalence or incidence among some ethnic minority groups, particularly Black and South Asian children. These patterns were often linked to socioeconomic disadvantage and differences in how families accessed or were referred to services.

Swedish studies consistently found higher ASD prevalence among children of immigrant parents especially those from non-Northern European regions. These groups were more likely diagnosed as ASD with intellectual disability or more severe forms of autism.

Conversely, the Dutch study reported a lower overall incidence of ASD among children of immigrant origin. This was mainly due to fewer diagnoses of Asperger syndrome and PDD-NOS.

Across several studies, children whose families were immigrants from low and middle income countries showed lower recorded rates of high-functioning ASD but relatively higher rates of more severe ASD presentations.

**Conclusion:**

Ethnicity and migration-related differences in ASD prevalence varied across countries and diagnostic subtypes. These patterns appear to reflect differences in diagnostic practices, service access and sociocultural factors. Thus, ethnicity data and cross-national comparisons of ASD prevalence should be interpreted cautiously, considering contextual and methodological factors.

